# The Role of Diet in Children with Psoriasis: Emerging Evidence and Current Issues

**DOI:** 10.3390/nu15071705

**Published:** 2023-03-31

**Authors:** Edoardo De Simoni, Giulio Rizzetto, Elisa Molinelli, Irene Capodaglio, Annamaria Offidani, Oriana Simonetti

**Affiliations:** 1 Clinic of Dermatology, Department of Clinical and Molecular Sciences, Polytechnic University of Marche, 60126 Ancona, Italy; 2 Hospital Cardiology and UTIC, Ospedali Riuniti di Ancona, 60126 Ancona, Italy

**Keywords:** psoriasis, pediatric population, Mediterranean diet, gluten-free diet, low-calorie diet

## Abstract

Psoriasis is an immune-mediated inflammatory systemic disease with skin tropism and chronic relapsing course; it is associated with an increased cardiovascular risk and with many metabolic comorbidities, emerging during childhood in 22–33% of cases. Diet influences the presentation and the clinical course of inflammatory diseases, including psoriasis; in particular, it was shown that a Mediterranean, gluten-free, or low-calorie diet may positively affect disease control in adult patients with psoriasis and adequate pharmacological therapy. These three dietary regimens may play a role also in children with psoriasis. It has been demonstrated that pediatric psoriasis is associated with psychological stress, celiac disease, and obesity, which may be positively influenced by these dietary regimens, respectively. Therefore, the expertise of multiple health figures (gastroenterologists, nutritionists, pediatricians, dermatologists) is required to plan a tailor-made dietary strategy, ensuring good growth, through an adequate intake of essential micro- and macronutrients and, at the same time, impacting the pro-inflammatory biochemical profile and on the associated cardiovascular risk of psoriasis disease.

## 1. Introduction

Psoriasis is an immune-mediated inflammatory systemic disease with skin tropism and a chronic relapsing course [1,2]. It affects about 1–2% of the population in the world and in 22–33% of cases starts in childhood [3]. Despite its unknown etiology, psoriasis is characterized by an interaction between multiple genetic and environmental risk factors [1,2].

Psoriasis is currently regarded as a systemic low-grade inflammation supported by a variety of cytokines, chemokines, and other inflammatory mediators including tumor necrosis factor (TNF)-α, interferon (IFN)-γ, interleukin (IL)-17, IL-22, IL-23, and IL-1β [4,5]. Patients with moderate-to-severe psoriasis show subclinical inflammation in the liver, joints, and tendons, in addition to significantly increased global arterial and subcutaneous inflammation [6,7,8,9]. In fact, systemic elevations in plasma levels of cytokines promote chronic subclinical inflammation associated with arthritis, cardiovascular comorbidities, and metabolic syndromes, such as obesity, dyslipidemia, diabetes, and atherosclerosis [10]. These associations have been also shown in psoriatic children. Among risk factors of psoriasis, overweight or obesity exert a main role in the development of the disease [11,12]. At the molecular level, leptin, a protein with a dual role as a hormone and as a cytokine, could have a key role in the relationship between obesity and psoriasis. In fact, leptin promotes the inflammatory response, highlighting that adipose tissue, besides the underlying low-grade inflammation, secretes immunomodulatory molecules, primarily impairing Th2 responses, while reinforcing Th1 inflammatory responses [13]. Elevated leptin levels are found in the majority of obese patients, suggesting a state of leptin resistance [14,15]. Moreover, psoriasis in children is associated with type 2 diabetes mellitus [16,17]. In particular, a recent study observed insulin resistance, a known condition that can lead to type 2 diabetes mellitus, in 16 out of the 60 psoriatic children recruited (27%), where 12 (75%) were overweight or obese and 14 (88%) had central obesity [18]. Previous studies also demonstrated that psoriasis is associated with oxidative stress in adults and in pediatric patients, corresponding to a significant increase in biochemical markers of oxidative stress of plasma lipids and lipoproteins, an imbalance of antioxidant enzymes, such as paraoxonase 1 [19,20], and an increase in pro-oxidant enzymes with a significant increase in the expression and activity of myeloperoxidase [21,22].

Therefore, it is very important to intervene early in metabolic diseases to prevent psoriatic skin manifestations, especially in pediatric patients with a family history of psoriasis [23]. Dietary, nutritional, and lifestyle choices exert key roles in health management and in the prevention of obesity and obesity-related diseases, including cardiovascular comorbidities. Obesity has been recognized as one of the main risk factors for the development and progression of psoriasis. On the other hand, weight loss regimens and dietary interventions have been demonstrated to be associated with beneficial results in the course of the disease [24]. A growing attention is devoted to gene–diet interactions in relation to obesity and other dysmetabolic diseases, representing an interesting field of application for innovative nutritional interventions in order to change health behaviors and prevent the development of chronic diseases associated with inflammation and oxidative stress. Both prenatal and infancy stages are considered the key steps in the development of childhood obesity, leading to the concept of the “first 1000 days” [21]. In detail, from the period of conception to 2 years of age, nutrition is considered a critical point that can trigger the onset and development of pathophysiological alterations leading to childhood obesity. At the molecular level, it has been demonstrated that epigenetic factors, including dietary factors in the maternal diet, can regulate gene expression at the transcriptional (via histone modification, DNA methylation) and post-transcriptional levels (via microRNAs and long non-coding RNAs) [25,26,27,28]. During neonatal age, both breastfeeding and complementary feeding are recognized as factors influencing the risk of developing obesity. Breastfeeding is associated with a 13% reduction in overweight or obesity, and the preventive effect on the risk of late obesity also depends on the duration of breastfeeding [29]. During childhood, eating habits, portion size choices, and dietary patterns appear to be predictive of later overweight and obesity rather than individual dietary nutrients alone [28]. Among possible determinants of obesity, a potential role is exerted by the infant gut microbiome. Human studies and advances in gene sequencing technologies have yielded evidence of the associations between gut microbiota and infant weight status [30].

It should be emphasized that obese children are more likely to become obese adults, and therefore interventions whose aims are to reduce the risk of such imprinting occurring should be focused on very early.

Mediterranean, gluten-free, or low-calorie diet and adequate pharmacological therapy may positively affect disease control in adult patients with psoriasis [31]. On the other hand, unbalanced dietary habits are more common in patients with psoriasis and may further worsen the pro-inflammatory milieu below the disease [31]. In detail, previous studies demonstrated that, compared to healthy controls, patients with psoriasis have higher intakes of total fat and simple carbohydrates and lower intakes of proteins, complex carbohydrates, monounsaturated fatty acids, n-3 polyunsaturated fatty acids (PUFAs), vegetables, and fibers [28,32].

Despite the incidence of psoriasis in children being 41/100,000 children-year, the impact of diet on disease control in the pediatric population has not been fully evaluated: in fact, few studies were conducted and data from the adult population are usually translated to children [33].

This narrative review aims to analyze the role of diet in patients with psoriasis, in particular in children, summarizing the biological and clinical evidence, and providing nutritional recommendations.

## 2. Materials and Methods

A literature search was performed on 2 September 2022 in Medline (PubMed); the keywords, alone or in combination, were “psoriasis”, “children”, “pediatric patients”, “diet”, “food”, and “nutrition”. Observational studies on pediatric patients and translational research were included. A time limit was not considered in the choice of studies. Only English-language studies were included.

## 3. Results

### 3.1. Mediterranean Diet

The Mediterranean diet (MD) is a dietary pattern found in olive-growing areas of the Mediterranean region and described in the 1960s and beyond [34]. Despite the controversial definition, MD is typically rich in fruits, vegetables, whole grains, beans, nuts, and seeds. Furthermore, it includes low to moderate amounts of fish, poultry, and dairy products, and little red meat [34]. In this diet, olive oil represents the most important source of monounsaturated fats.

MD is associated with the prevention of dysmetabolic, cardiovascular, and chronic inflammatory diseases [35]. In fact, its positive impact on adults with psoriasis has been proposed. Barrea et al. conducted a cross-sectional case-control observational study including 62 adult patients with mild-to-severe psoriasis and 62 healthy controls. They evaluated the adherence to the MD with a score (PREDIMED questionnaire) and assessed its impact on the activity of the disease in terms of the Psoriasis Area and Severity Index (PASI) score. The PREDIMED questionnaire includes 14 items about behaviors consistent with the MD. The authors demonstrated a lower adherence to the MD in patients with psoriasis and an inverse correlation between adherence to MD and activity of disease (*p* < 0.001) [36].

Similarly, Phan and colleagues prospectively evaluated the adherence to MD in 3557 adult patients with psoriasis thanks to a similar score (MEDI-LITE); among patients with severe disease (*n* = 878), a significant inverse association between adherence to MD and severity of disease was demonstrated [17].

Both these findings may support the hypothesis that MD is associated with less severe psoriasis and that the nutrients with anti-inflammatory properties included in MD may affect the course of disease and synergize with pharmacological treatments. For instance, vegetable dietary factors are a source of phytonutrients (polyphenols, flavonoids) that exert potentially anti-inflammatory effects [37] Specifically, MD is rich in hydroxycinnamic derivatives, quercetin, resveratrol, oleuropein, and hydroxytyrosol, which act as antioxidants and anti-inflammatories, blocking the overproduction of reactive oxygen species (ROS). The pathophysiological mechanism of the inflammatory setting in obese patients is based on the increased expression of adipokines such as TNF-α, which are responsive to the oxidative state, through the activation of nuclear factor-κB (NF-κB). In addition, TNF-α is active by blocking AMP-activated protein kinase (AMPK), which plays a role in maintaining the homeostasis of the inflammatory response. Finally, polyphenols can reduce the activity of nicotinamide adenine dinucleotide phosphate (NADPH) and oxidase (NOX), controlling the obesity associated-inflammation [37].

No studies have been conducted to demonstrate this association in psoriatic children. However, they are known to benefit from MD adherence in terms of quality of life and cardiovascular risk [38]. In fact, a systematic review evaluated the association between MD adherence and health-related quality of life (HRQoL) in children and adolescents: 11 studies with a total of 6796 patients (older than 6 years and younger than 18 years) were included. The HRQoL questionnaire explored physical and psychological well-being, parent relations and autonomy, and the school environment. All studies analyzed the association with linear regression and eight used adjusted models: among the latter, five found a significant positive association between DM adherence and HRQoL (with β values ranging from 0.13 to 0.26), two found a non-significant positive relationship, while one found a negative association. Recently, a positive correlation between MD adherence and HRQoL in both children and adolescents was suggested [39,40].

Higher rates of adherence to MD are observed during pregnancy and then gradually decrease during lactation [11,12]. Poor adherence is observed during preschool and school age, but it increases positively during adolescence [12]. These results are helpful for identifying who is at the highest risk of developing unhealthy eating habits, which should be targeted with nutritional re-educational programs.

### 3.2. Gluten-Free Diet

A gluten-free diet (GFD) is an alimentary regimen not containing gluten, a protein found in wheat, rye, barley, and oats; many foods, such as bread, pasta, pizza, and cereals, contain gluten. GFD is indicated in patients with celiac disease (CD), a systemic immune-mediated disorder triggered by dietary gluten in genetically susceptible persons. CD is characterized by a broad range of clinical presentations and variable damage to the small-intestinal mucosa [41].

Although gluten intake does not represent a risk factor for psoriasis, a higher risk of psoriasis in patients with CD (OR: 1.8) and, similarly, an increased risk of CD in patients with psoriasis (OR: 2.16) were observed in a meta-analysis [42]. For example, in a study enrolling 28,959 patients with CD and 143,910 healthy controls, CD was found to be a risk factor for future psoriasis (Hazard Ratio [HR]: 1.72; 95% CI: 1.54–1.92); this result was even more robust in the pediatric population, which corresponded to about 40% of the enrolled population (HR: 2.05; 95% CI: 1.62–2.60) [43].

In addition, antigliadin IgA antibody levels, which represent a diagnostic criterion for CD, were also found to be higher in patients with psoriasis [44].

Anyway, studies on GFD in patients with psoriasis showed a clinical benefit in terms of disease control only for those with CD; for example, Michaëlsson et al. showed that adult patients with psoriasis and positivity for antigliadin IgA or IgG had a significant PASI score decrease after three months of GFD (reduction in PASI from 5.5 ± 4.5 to 3.6 ± 3.0); after returning to a non-GFD, 60% of patients had worsening of psoriasis symptoms. Instead, in patients with antigliadin IgA or IgG in the range, the activity of psoriatic disease was not affected by a GFD [45].

The role of gluten in the diet of children with psoriasis has not been directly investigated, but, on the basis of the biological connections between these immune-related disorders, children with psoriasis and bowel symptoms suggestive of CD should be referred to a gastroenterologist to exclude gluten-related diseases. At present, GFD seems not to have a role either in adult or pediatric patients with psoriasis and without CD. Moreover, reduced intake of micronutrients and fiber as well as enhanced consumption of fat and simple carbohydrates has consistently been reported in several gluten-free products [46,47].

Nowadays anti-gliadin antibody(AGA)-testing has been replaced by more accurate serological assays largely because of poor specificity. The igA-TG2 antibody is the preferred single test for the detection of CD at any age [48]. The use of GFD raises many doubts as Owczarczyk-Saczonek explained in her publication [49]: one of the best-known studies reported a reduction in psoriatic lesions us AGA-positive patients after GFD, while there were no changes in AGA-negative patients. On the other hand, AGA-positive patients worsened again with the reintroduction of gluten in the diet. However, AGA-positivity is common, especially among the older population [50].

A meta-analysis recently showed a significant association between psoriasis and CD, suggesting that the gut–skin axis may drive inflammatory processes. Gut inflammation results in increased permeability to exogenous antigens, leading to an inflammatory response that is first local and then systemic, stimulating the skin manifestations of psoriasis [51]. On the other hand, a study of 85,185 female psoriatic patients and 85,324 with PsA showed no significant effect of increased gluten intake on psoriasis and PsA [52]. In conclusion, GFD should only be adopted in patients with confirmed CD. Therefore, it is important not to underestimate patients’ dyspeptic complaints and, if indicated, to perform diagnostic tests, in particular in first-degree relatives with CD or in patients with active gastrointestinal symptoms [25].

### 3.3. Low-Calorie Diet

A low-calorie diet (LCD) is an alimentary regimen characterized by caloric intake restriction and is recommended for patients with excess body weight [1].

Data in adult obese patients with psoriasis suggest a positive association between LCDs, weight reduction, and better control of psoriasis [1]. In fact, a meta-analysis of six randomized control trials demonstrated that obese patients who received an LCD were significantly more likely to achieve a 75% or greater reduction in PASI score from baseline (PASI75; Relative Risk [RR]: 1.47, 95% CI: 1.27–1.69) and to have a reduction in mean PASI score of −2.59 (95% CI: −4.09, −1.09) [46].

Furthermore, LCD diets have been linked to a better response to the treatment of systemic psoriasis, making them a useful adjunctive treatment; in fact, it was demonstrated that obese patients with psoriasis, who began a traditional systemic therapy, experienced a greater therapeutic response in terms of PASI when subjected to an LCD compared with the relative control [53].

This evidence may be explained by preclinical data demonstrating that obesity and overweight are associated with an increase in inflammatory cytokines (e.g., IL-6 and TNF-), which represent key mediators in psoriasis [4,5,13].

Also in the pediatric population, obesity and overweight, whose prevalence is growing (from 4% in 1975 to 18% in 2016, for a total of 380 million children), are associated with psoriasis [11,54,55]. An international cross-sectional study found that the OR of obesity, defined as the 85th percentile of body mass index (BMI), in 409 children with psoriasis and 205 healthy controls was 3.60 (95% CI: 1.56–8.30) and 4.92 (95% CI: 1.96–9.39) for mild and severe disease, respectively, compared with controls [53]. Similarly, in a case-control study of 20 children aged 5–15 years old with moderate-to-severe plaque psoriasis, the probability of being obese was four times higher than the 27 healthy controls (OR: 4.4; 95% CI: 1.2–15.6) [56]; in addition, the probability of metabolic syndrome was also higher in the psoriatic group. Furthermore, this last evidence was also prospectively confirmed in a study comparing 20 children aged 9–17 years with psoriasis or psoriatic arthritis: it demonstrated that 30% of children with psoriasis met the criteria for metabolic syndrome, compared with 5% in the control group [56]. Moreover in psoriatic children, an increase in cholesterol associated with HDL, LDL, and VLDL as well as a significant decrease in the apo-protein content in all lipoprotein fractions has been observed in psoriatic children with respect to controls [57,58] A recent report showed an imbalance of Myeloperoxidase and Paraoxonase-1 suggesting in psoriatic children high oxidative stress in serum lipoproteins [21] as observed in other chronic inflammatory diseases [59].

In conclusion, on the basis of data from the adult population and in light of the strong association between obesity and psoriasis in children, despite the lack of evidence about the impact of LCDs on psoriasis control, LCD should be indicated in obese children, especially to control the cardiovascular risk. Data from observational studies in children with psoriasis are awaited.

## 4. Discussion

MD, GFD, and LCD have been demonstrated to positively impact the clinical history of psoriasis in selected adult patients. Many observational and interventional studies have been conducted in these patients: they showed that a specific alimentary regimen determines a decrease in PASI score on the basis of an accurate patient selection; in particular, LCD has a role in obese patients with psoriasis and GFD in patients with psoriasis and CD or positivity to an antigliadine test. Furthermore, these diets have shown also to decrease cardiovascular risk, which is known to be increased in patients with psoriasis [1,10].

In one-third of patients, psoriasis is diagnosed in childhood and negatively affects the quality of life [11]; furthermore, also in the pediatric population, it is associated with metabolic comorbidities and an increased cardiovascular risk, which may lead to a major cardiovascular event in adulthood.

Anyway, no data about the impact of a dietary regimen in children with psoriasis have been collected and, in the absence of clinical evidence, merely transferring evidence from adults to children requires the demonstration of a strong biological and clinical rationale, because children cannot simply be considered small adults.

**Figure 1:**
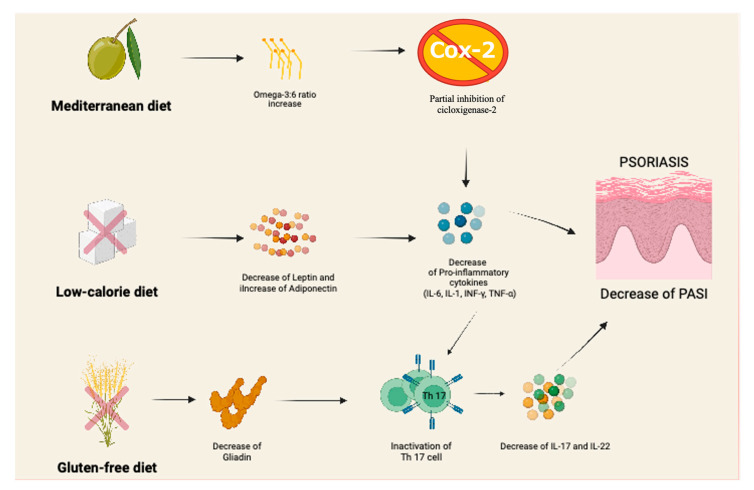
Hypothetical mechanisms of dietary regimens in children with psoriasis. Gluten-free diet mechanisms are considered only if patients with Celiac Disease.

Figure 1 summarizes potential mechanisms involved in the protective effect exerted by different diets against inflammatory mechanisms in psoriasis. As far as MD is concerned, unsaturated fats may affect immune-metabolic pathways [60]. In fact, oleic acid, a monounsaturated fat found in olive oil, protects cell membranes from harmful oxidative effects, and many polyunsaturated fatty acids, such as omega-3 and omega-6, are involved in the synthesis of anti-inflammatory and pro-inflammatory compounds, respectively [13]. In particular, omega-3 fatty acids compete with arachidonic acid to bind to COX-2 (cyclooxygenase-2), contributing to an anti-inflammatory effect.

The ratio of omega-3 to omega-6 family fatty acids should be balanced, and patients with inflammatory disease should conduct a diet rich in omega-3 fatty acids, while omega-6 acids should be limited [30,61,62,63]. In the study by Barrea et al., an analysis of dietary fatty acid content was performed by evaluating seven daily food rations of 41 men with psoriasis compared with a control group. In patients with psoriasis, a higher intake of omega-6 acids and a lower intake of omega-3 acids were shown compared with the control group. In addition, an abnormal ratio of omega-6 to omega-3 acids was correlated with a higher PASI [64,65].

In regard to LCD, adipose tissue has been demonstrated to secrete pro-inflammatory cytokines (adipokines) that negatively affect the course of psoriasis [30]; for example, the increased levels of phospholipase A2, arachidonic acid and its metabolites found in psoriatic plaques may increase epidermal cell proliferation and inflammation [45]. Possible reasons for the psoriasis improvements achieved with LCD focused on weight loss may be due to a reduction in abdominal fat and adiposity, resulting in lower adipokine levels and decreased inflammation underlying the psoriatic march. Although the relationship between psoriasis severity and various serum levels of adipokines remains unclear, Nakajima et al. found that adiponectin, a serum adipokine, correlated positively with PASI scores, as did IL-22 [66]. The mechanism behind obesity exacerbating psoriasis could therefore be due to adipokines inducing an increase in Th-17-related cytokines.

Lastly, as far as GFD is concerned, the underlying mechanism of action of PASI decrease after GFD is not yet fully understood; anyway, in patients affected by CD, it has been demonstrated that gliadin induces sensitization of T cells, which may play a role in the pathogenesis of psoriatic lesions [67].

In conclusion, these three dietary regimens may play a role also in children with psoriasis; in fact, it has been demonstrated that pediatric psoriasis is associated with psychological stress, CD, and obesity, which may be positively influenced by MD, GFD, and LCD, respectively. An intriguing biological rationale has been demonstrated and clinical data from observational studies and clinical trials are awaited.

Meanwhile, great caution is required when prescribing a dietary regimen to a child; in fact, drastic changes in diets should be avoided to prevent nutritional deficiencies, especially with respect to essential nutrients, which are paramount for children’s growth [68]. In this regard, vegetarian and plant-based diets do not provide an adequate nutritional intake and are associated with higher odds of underweight in children [69]. More specifically, an adequate intake of minerals and antioxidants should be adequately administered in pediatric patients. According to the European Food and Safety Authority (EFSA), adequate intakes for the main minerals are as follows: copper 0.7 mg/day for children from 1 to 3 years, 1 mg/day for children from 3 to 10 years, and 1.3 mg/day for children from 10 to 18 years; iron, 11 mg/day in infants from 7 to 11 months, 7 mg/day in children from 1 to 6 years, and 11 mg/day in children from 7 to 11 years; zinc 2. 4 to 11.8 mg/day in infants from 7 months and older, and for children. With regard to adequate antioxidant intakes, α-tocopherol should be consumed 6 mg/day for children from 1 to 3 years, 9 mg/day for children from 3 to 10 years, and 13 mg/day for boys and 11 mg/day for girls from 10 to 18 years; carotenoids 2.8–11 mg/day for children from 5 to 17 years. Daily intake of vitamins includes vitamin D 15 μg/day from 1 to 17 years and 10 μg/day from 7 to 11 months; vitamin C 20 mg/day from 1 to 3 years, and 90 mg/day for 15–17 years; vitamin A 190 µg retinol equivalent (RE)/day from 7 to 11 months, 580 µg RE/day in boys from 15–17 years, and from 250 to 750 µg RE/day for infants and children [70].

In light of these values, we recommend adequate antioxidant intake to prevent or reduce oxidative stress. Again, there is a lack of specific studies for pediatric age, so at the moment, we recommend following EFSA guidelines for daily intakes of antioxidants. Further studies are needed to assess whether increased dietary antioxidant intake can further improve oxidative stress in pediatric patients with psoriasis. However, we strongly suggest that the intake of vitamins A, C, D, and E in children should be sufficient to ensure their regulatory functions. Furthermore, we report the daily intakes of nutrients in pediatric patients as a guideline: carbohydrates from 45 to 60% for children older than one year of age; fat intake can gradually be reduced from 40% in the 6–12-month period to 35–40% in the 2nd and 3rd year of life, and proteins about 0.66 g/kg body weight per day [70].

Among minerals, selenium plays an important role in the antioxidant system, as was demonstrated in the mouse model treated with topical selenium nanoparticles. In a recent study, Gangadevi et al. reported that selenium nanoparticles lead to a significant reduction in the severity of psoriasis in mice by modulating inflammatory and keratinocyte proliferation mediators. In the future, the combination of an antioxidant diet and topical application of selenium could be a valuable support in reducing oxidative stress in humans as well [71].

Recently, Zanesco et al. [72] proposed a project to evaluate the impact of MD and time-restricted eating in psoriasis, called the diet and psoriasis project. Although this study will be conducted on patients ≥18 years old, some aspects can also be transferred to the pediatric population. Among the micronutrients, flavonoids contribute to the control of the inflammatory cascade by reducing the production of TNF-a, IL-8, IL-6, and IL-1b [73]. Vitamin C and vitamin A act as antioxidants and reduce the inflammatory process [74]. Vitamin E and polyphenols are associated with an improvement of the endothelium and a beneficial effect on lipid metabolism, reducing LDL oxidation and inflammatory mediators [75]. Vitamin D Eicosapentaenoic acid (EPA) and docosahexaenoic acid (DHA) act as modulators of inflammation by competing with omega-6 substrates and reducing the production of eicosanoids [76]. DHA and EPA also reduce the action of NF-kB, with decreased IL-6 and TNF-a, and downregulate the expression of ICAM-1 and VCAM-1 [77]. Vitamin D is also important in controlling keratinocyte proliferation, maintenance of the skin barrier, and the inflammatory response [78].

Fiber also acts on metabolism, aiding intestinal transit and promoting the selection of a eubiotic microbiota, with a higher ratio of Firmicutes and Bacteroides compared to Proteobacteria, capable of producing short-chain fatty acids with regulatory action on the inflammatory response [79,80]. Finally, correct diet habits could influence not only the microbiota composition but also the microbiome composition [81].

Therefore, the expertise of multiple health figures (gastroenterologists, nutritionists, pediatricians, dermatologists) is required to plan a tailor-made dietary strategy, aiming at ensuring good growth, the essential micro- and macronutrients and, at the same time, impacting the pro-inflammatory biochemical profile and the associated cardiovascular risk. Moreover, this approach should be integrated with indications for a healthy lifestyle including physical activity, which has been demonstrated to impact the clinical course of psoriasis [82].

## 5. Conclusions

Diet and nutrition for patients with psoriasis represent a complex field and no data are available from the pediatric population. However, some evidence about MD, GFD, and LCD may be transferred from adults to children in light of a strong preclinical rationale and clinical data highlighting the association between pediatric psoriasis, psychological stress, CD, and obesity. This nutritional effort should be combined with the optimization of pharmacological therapy and guided by specialists, who may consider the history of psoriasis and the energy requirements for children’s growth.

## Data Availability

All data in the manuscript.
